# The antihelminthic drug niclosamide effectively inhibits the malignant phenotypes of uveal melanoma *in vitr*o and *in vivo*: Erratum

**DOI:** 10.7150/thno.47923

**Published:** 2020-06-10

**Authors:** Jingfeng Zhou, Bei Jin, Yanli Jin, Yizhi Liu, Jingxuan Pan

**Affiliations:** State Key Laboratory of Ophthalmology, Zhongshan Ophthalmic Center, Sun Yat-sen University, 54 S. Xianlie Road, Guangzhou, 510060, China

In our article 1, there were one misplaced image in Fig. 2C and Fig. 6B, respectively. The corrected version of Figure 2C and Figure 6B are provided here.

In addition, the description of the whole section regarding all the supported grants originally stated in the acknowledgements in the initially published version of this article 1 is replaced with: The study was supported by grant (Grant 2015A030312014 to J. Pan) from Natural Science Foundation of Guangdong Province.

The corrections made in this erratum do not affect the original conclusions. The authors apologize for any inconvenience or misunderstanding that these errors may have caused.

## Figures and Tables

**Figure 2C:**
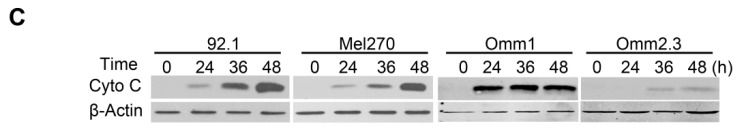
UM cells were exposed to niclosamide for 48 h, Western blot analysis of cytochrome c in the cytosolic extracts.

**Figure 6B:**
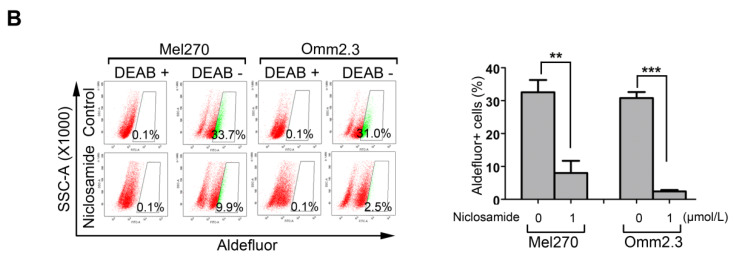
Mel270 and Omm2.3 cells were treated with niclosamide (1 μmol/L) for 48 h, and the percentage of Aldefluor^+^ cells was determined by flow cytometry. Representative flow cytometry data and bar graph with SD from 3 independent experiments are shown. **, p< 0.01; ***, p< 0.001, Student's* t* test.

## References

[1] Zhou J, Jin B, Jin Y, Liu Y, Pan J (2017). The antihelminthic drug niclosamide effectively inhibits the malignant phenotypes of uveal melanoma in vitro and in vivo. Theranostics.

